# Complexity and Simplification in Understanding Travel Preferences Among Tourists

**DOI:** 10.3389/fpsyg.2019.02302

**Published:** 2019-10-17

**Authors:** Torvald Øgaard, Rouven Doran, Svein Larsen, Katharina Wolff

**Affiliations:** ^1^Norwegian School of Hotel Management, Faculty of Social Sciences, University of Stavanger, Stavanger, Norway; ^2^Department of Psychosocial Science, Faculty of Psychology, University of Bergen, Bergen, Norway

**Keywords:** tourist role orientation, destination valuations, destination perceptions, revisit intentions, novelty, familiarity

## Abstract

Travel preferences are complex phenomena, and thus cumbersome to deal with in full width in diagnostic and strategic planning processes. The aim of the present investigation was to explore to what extent individual preferences can be simplified into structures, and if tourists can be grouped into preference clusters that are viable and practically applicable for tourism planning. Building on prior studies that have validated survey instruments designed to measure different tourist role orientations, we used a factor analytical approach to develop a simplified structure of individual preferences, and a standard clustering technique for grouping tourists into preference clusters. Further analyses indicated that preference clusters based on reduced factor preference-data are to some extent related to context-specific valuations, perceptions, and revisit intentions; however, the magnitude of differences between groups was rather small. Overall findings provide reason to suggest that the identified preference clusters are insufficient when it comes to explaining variability in which aspects tourists emphasize as part of their vacation. Possible managerial implications and methodological limitations of the present investigation are noted.

## Introduction

A fundamental need for any business or public planning in the tourism sector is to map heterogeneity among people (Dolnicar, 2008) and to understand the psychological processes involved in the construction of the tourist experience (Larsen, 2007). Products and services offered within the sector are complex and individual preferences may comprise one or more of the following elements: nature, culture, food, activities, social interaction etc. The experiences of a destination will comprise an even more complicated process involving both elements that a person has preferences for, and numerous additional expected and unexpected exposures to elements that may be important for the total experience. Planners and managers in the tourism industry who are eager to make evidence-based decisions may therefore benefit from searching, surveying, incorporating, synthesizing, and presenting such information; for a more broad discussion on opportunities for tourism marketing research, see Dolnicar and Ring (2014).

While information about preferences can be available from public source materials, such as the number of tourists visiting a specific destination at a given moment in time, further information that is more detailed can be collected and then be evaluated by the tourism industry itself. For this purpose, there have been numerous market segmentation studies to suggest a wide range of objective and subjective measures aimed at categorizing individuals into groups (Díaz-Martín et al., 2000; Frochot and Morrison, 2000). The groups developed in these studies more often than not were data-driven, and in spite of them producing adequate segmentation of specific groups in particular contexts and at a given point in time, the resulting segments were less comparable across studies; thus, limiting cumulative knowledge development. And even though these studies quite often were able to simplify the data-structure, the obtained structure was typically not standard and transferable across studies, and the stability of the segments was not a major concern (Dolnicar, 2008).

An inspection of the existing literature suggests that efforts to simplify and structure data addressing preference heterogeneity in tourism settings have progressed along different lines. Some studies have focused on simplifying the structure of the data by reducing its complexity through collapsing the number of independent elements into broader categories (e.g., Mo et al., 1993; Jiang et al., 2000; Gnoth and Zins, 2010), whereas other studies have attempted to group together travelers based on homogeneity alongside individual preferences (e.g., Yiannakis and Gibson, 1992; Gibson and Yiannakis, 2002). The empirical investigation reported as part of this paper sequentially addresses both of the aforementioned approaches. We first simplify the data structure of the preference-data through a factor analytic approach, and then group the tourists into clusters based on the simplified preference-data. These procedures are followed by a validation of the clusters by evaluating if they are useful for explaining context-related valuations, perceptions, and revisit intentions; for reviews on psychological and sociological approaches to understanding travel motivations, see Heitmann (2011) and Dann (2018).

One of the first attempts to describe and categorize tourists stems from Cohen (e.g., 1972) who distinguished four types based on their relationship with the tourism industry and the host country.^1^ On one hand, there are individuals who engage in institutionalized arrangements that have a strong need for familiarity, low interest for adventures, and little contact with local culture or hosts (described as the “organized mass tourist”), whereas others in this category are less rigid insofar that they allow some greater room for personal choice (described as the “individual mass tourist”). On the other hand, there are individuals traveling independently that restrict any meeting with the industry unless unavoidable (described as the “explorer”), and those who refuse contact with the industry altogether, put as much distance between themselves and their familiar home environment as possible, and become part of the host culture (described as the “drifter”). It is the extent by which individuals seek to move away from their familiar (social and cultural) environment that shapes their relationship with the tourism industry (Cohen, 1972) and their respective mode of experience (Cohen, 1979). While there is empirical evidence to support the view that tourists can be clustered according to the aforementioned types (Snepenger, 1987), some scholars argued that these are merely examples of an even larger number of possible roles individuals can take on when traveling for leisure purposes (Yiannakis and Gibson, 1992).

The International Tourist Role Scale (ITR; Mo et al., 1993) operationalizes the abovementioned taxonomy by distinguishing individuals based on their preferences on a novelty-familiarity continuum. The scale comprises of 20 items that aim at measuring one of three distinct dimensions used to differentiate tourists in an international context. One so-called “destination-oriented” dimension that measures the degree to which tourists prefer novel versus familiar holiday destinations. Another so-called “travel services” dimension that attempts to measure the degree to which tourists prefer institutionalized arrangements at their holiday destination. As well as an additional so-called “social contact” dimension, that seeks to assess the degree to which tourists prefer contact with local residents at their holiday destination. Previous research has reported empirical evidence in favor of a three-dimensional structure proposed by the original 20-item version (Mo et al., 1994) as well as by a revised 16-item version (Jiang et al., 2000).

With the above studies having focused upon confirming the psychometric properties of the ITR scale, others have demonstrated its predictive validity in explaining aspects of the tourist experience. For example, Wolff and Larsen (2019) showed that people who prefer traveling to familiar rather than unfamiliar destinations tend to be less likely to show an interest in trying out new food. The same study found that people who like better to avoid institutionalized forms of tourism were on average more interested in trying out new food than those favoring the opposite; the same applied to those who prefer seeking out social encounters with local people and culture. Others studies indicate that individual differences on one or more of the dimensions outlined by the ITR scale can be associated with social interactions experienced by guests (Basala and Klenosky, 2001), risk perceptions about international tourism (Lepp and Gibson, 2003), importance ascribed to vacation activities (Keng and Cheng, 1999), and preferred social contacts with hosts (Fan et al., 2017). Further research suggests that the degree to which individuals emphasize either one of the proposed dimensions can vary as a function of cultural values (Gnoth and Zins, 2010).

### Research Aims

Previous studies attempting to validate theoretically developed tourist typologies have employed different methods including quantitative approaches (e.g., Mo et al., 1993) as well as qualitative approaches (e.g., Uriely et al., 2002). The present paper draws upon the idea that tourists differ in their preferences, and that considering these differences can yield useful information for planners and managers in the tourism industry. Consequently, the following section reports on an empirical investigation that set out to explore the relationship between travel preferences, destination valuations, destination perceptions, and revisit intentions. Research on these aspects can provide useful insights for at least two reasons: to gain some better understanding about what tourists search for in their vacation and to provide some guidance for those interested in developing products and services that address these preferences at a given destination.

## Materials and Methods

### Participants

The analyzed data comprises the responses from *N* = 2041 individuals who were visiting Western Norway during the summer months. Norway as a holiday destination tends to attract visitors from abroad who seek to experience both nature and culture, preferably in combination (Innovation Norway, 2018). Participants were between 18 and 82 years (*M*_age_ = 37.06, *SD*_age_ = 14.88), were on trips that lasted between 1 day and more than 2 years (*Mdn*_days_ = 12), and the most prevalent day for filling in the questionnaire was at day five during their current trip (*Mdn*_days_ = 5). They differed in a variety of different ways including their gender (47.8% men, 52.0% female), accommodation (camping facility 15.0%, private pension 12.7%, HI hostel 10.8%, hotel 28.3%, cruise ship 8.7%, not specified 22.6%), continent (Europe 67.6%, North America 12.8%, South America 2.3%, Oceania 5.0%, Asia 7.5%, Africa 0.7%), and tourism type (international 94.9%, domestic 3.8%).^2^

### Procedure

To recruit participants, research assistants first approached potential participants at well-known sightseeing spots, either in person or in groups, accompanied by the question of whether they are on vacation. In a second step, research assistants invited them to participate in a study on tourist experiences granted that they affirmed positive to the prior questions. A written statement on the questionnaire informed participants that there are no right (or wrong) answers, and that each of the answers would be handled confidentially. Informed consent was implied by the completion of the questionnaire.

Each participant responded to a survey distributed as part of a larger research project investigating various aspects of the tourist experience (paper questionnaire, four pages long, English language). Items included in the survey focused upon a broad range of topics relevant to illuminate experiences, perceptions, and behaviors in tourism settings; yet, this paper focuses exclusively on the aforementioned socio-demographic characteristics (see above), as well as on measures pertaining to travel preferences, destination valuations, destination perceptions, and revisit intentions (see below).

### Materials

Travel preferences were measured with a revised 16-item version (Jiang et al., 2000) of the ITR scale (Mo et al., 1993, 1994). The revised scale comprises three subscales that measure reported preferences with regard to the destination itself, travel arrangements, and socio-cultural aspects linked to traveling. Respondents were asked to indicate their agreement to each of the item statements presented on a 7-point Likert-type scale (1 = Strongly disagree, 7 = Strongly agree; see Table 1).

**TABLE 1:** Measurement items for travel preferences.

**Item label**	**Item statement^a^**
O1	I prefer to start a trip with no preplanned or definite routes when traveling in a foreign country.
O2	I prefer to travel to countries where they have the same tourism infrastructure (such as highways, water supply, sewers, electric power, and communications systems) as in my country.
O3	I prefer to seek the excitement of complete novelty by engaging in direct contact with a variety of new and different people.
O4	I prefer to travel to countries where the culture is different from mine.
O5	I prefer to make no major arrangements through travel agencies when traveling in a foreign country.
O6	I prefer to travel to countries with well-developed tourism industries.
O7	I prefer to start a trip with no preplanned or definite routes when traveling in a foreign country.
O8	I prefer to travel to countries where there are restaurants familiar to me.
O9	If I find a place that particularly pleases me, I may stop there long enough for social involvement in the life of the place to occur.
O10	I put high priority on familiarity when thinking of destinations.
O11	I prefer to travel to countries where they have the same transportation system as in my country.
O12	I prefer not to be on a guided tour when traveling in a foreign country.
O13	I prefer to have little personal contact with the local people when traveling in a foreign country.
O14	I prefer to live the way the people I visit live by sharing their shelter, food, and customs during my stay.
O15	I prefer to have travel agencies take complete care of me, from beginning to end, when traveling in a foreign country.
O16	I prefer to make friends with the local people when traveling in a foreign country.

*Response scale anchored at 1 = Strongly disagree and 7 = Strongly agree. Instructions were “Please read the following statements and indicate to which degree you agree with these statements:” ^*a*^From the revised 16-item version of the ITR scale (Jiang et al., 2000).*

Destination valuations were measured with 13 item statements asking participants about how important various aspects relating to their current trip were when they initially bought the trip. For each statement, respondents were asked to rate the importance of the aspect when buying the current trip using a 7-point Likert-type scale (1 = Not important, 7 = Very important; see Table 2).

**TABLE 2:** Measurement items for destination valuations^a^ and destination perceptions^b^.

**Item label**	**Item statement**
TA1	Affordable price
TA2	Opportunity to experience nature
TA3	Opportunity for outdoor activities like hiking/boating
TA4	Opportunity to go fishing
TA5	Opportunity for cultural experiences like museums or concerts
TA6	Opportunity to visit exceptional landscapes
TA7	Opportunity to go shopping
TA8	Opportunity to try local food
TA9	Opportunity to enjoy good climate/clean air
TA10	Opportunity to meet the locals
TA11	That the trip does not harm the environment
TA12	That the trip does not disturb the local community
TA13	That the trip contributes to the local economy

*Item statements were the same for destination valuations and destination perceptions, but response scales and instructions differed in each case. ^*a*^Response scale anchored at 1 = Not important and 7 = Very important. Instructions were “How important were the following aspects when buying your current trip to Norway?” ^*b*^Response scale anchored at 1 = Not at all and 7 = Very much. Instructions were “To which degree did/does your current trip to Norway offer these aspects?”*

Destination perceptions were assessed using a similar approach as outlined above; that is, the same selection of items were rated on the extent to which the present trip to the country offered each respective aspect, which was again made based on a 7-point Likert-type scale (1 = Not at all, 7 = Very much; see Table 2).

All surveys contained the measures described above, yet one share of the distributed surveys (*n* = 1162) further included three items to measure revisit intentions. Respondents indicated the likelihood by which they – within the next 3 years – would repeat the current trip, revisit the same city, and/or revisit the same country. Higher scores on a 7-point Likert-type scale were taken as greater revisit intentions respectively (1 = Very unlikely, 7 = Very likely; see Table 3).

**TABLE 3:** Measurement items for revisit intentions.

**Item label**	**Item statement**
VI1	…that you will repeat the trip you are on now?
VI2	…that you will visit Bergen again?
VI3	…that you will visit Norway again?

*Response scale anchored at 1 = Very unlikely and 7 = Very likely. Instructions were “Within in the next 3 years, how likely is it…”*

### Analyses

The statistical analyses were performed with IBM SPSS Statistics v. 25 in the following order. First, we re-validated the factor structure of a revised version of the ITR scale. Second, we used the developed factor structure for grouping tourists into preference segments (clusters) and then we evaluated the concurrent validity of the groups by analyzing demographic differences between these segments. Third, we analyzed if (and how) the valuation of destination aspects varies between groups; the same analysis was run with destination perceptions and revisit intentions as dependent variables.

## Results

### Factor Analyses

Jiang et al. (2000) suggested three substantial factors addressing destination-oriented, socio-cultural, and travel arrangement aspects when people choose to go on vacation abroad. The Kaiser–Meyer–Olkin (KMO) measure of sampling adequacy was well above 0.5 and Bartlett’s test of sphericity was significant at *p* < 0.001, which indicates that the current data was suitable for factor analysis (see Field, 2018). Table 4 contains results from an exploratory factor analysis (principal axis analysis with direct oblimin rotation). The analyses produced a slightly diverging pattern with four factors having an eigenvalue greater than one. The fourth factor (eigenvalue 1.23), containing items O5, O12, and O15, split the travel arrangement factor in two. A parallel analysis (Horn, 1965) performed on 1000 random datasets with 16 variables resulted in an average eigenvalue of the fourth factor of 1.09, indicating that the eigenvalue of the fourth factor of the present set is marginally above a random value.

**TABLE 4:** Pattern matrix, exploratory factor analysis, principal axis, oblimin rotation, 16 items.

**Item label**	**Factor loading^a^**				
	**Destination-oriented**	**Socio-cultural**	**Travel arrangement-one**	**Travel arrangement-two**	**Communality**
O1	−0.181	0.116	**0.635**	0.148	0.494
O2	**0.674**	−0.064	−0.092	0.098	0.427
O3	0.000	**0.680**	0.031	−0.021	0.467
O4	−0.139	**0.496**	−0.047	−0.002	0.280
O5	0.045	0.080	0.130	**0.463**	0.253
O6	**0.638**	0.038	−0.161	0.034	0.372
O7	−0.027	0.069	**0.519**	0.068	0.296
O8	**0.581**	−0.039	0.214	−0.162	0.518
O9	0.126	**0.466**	0.150	0.013	0.282
O10	**0.491**	0.083	0.194	−0.208	0.411
O11	**0.770**	−0.023	0.045	0.040	0.599
O12	0.080	−0.021	0.023	**0.517**	0.248
O13	0.175	−0.112	0.264	−0.086	0.142
O14	0.000	**0.587**	0.010	0.035	0.358
O15^b^	−0.278	−0.042	−0.082	**0.468**	0.374
O16	0.051	**0.784**	−0.067	−0.020	0.576
Eigenvalue	3.301	2.627	1.381	1.230	

*KMO > 0.7. Barlett’s test of sphericity was significant at *p* < 0.001. Rotation converged in nine iterations. ^*a*^Boldface indicates factor loadings <0.40. ^*b*^Item that was reverse coded for the analyses.*

Table 4 shows that most items were well accounted for by the factor structure, with factor loadings above 0.40 on the main factor and comparatively low cross loadings. Item O13 was excluded from further analyses because only items with factor loadings greater than 0.40 were retained for more exploration. Item O15 showed a modest loading on the travel arrangement-two dimension, but also a non-negligible cross loading on the destination-oriented dimension. This item was retained for further analyses after an inspection of its corresponding Marker Index, which was above the recommended 0.40 threshold (Gallucci and Perugini, 2007).

Rather than unequivocally reproducing the factor structure reported by Jiang et al. (2000), the current data suggests that the scale could be developed further to improve fit to the data, particularly by splitting travel arrangement aspects in two factors. Including an extra factor would nevertheless only account for a marginal part of the total variance, whereas some of the common variance of this tentative factor may further be due to sample- or context-specific features. We therefore chose to keep the three-factor structure to allow direct comparisons with other studies that have utilized the scale. This means that we calculated average scores for each individual respondent on each of the three dimensions displayed in Table 5. Each item was weight equally (unit weights) and cross loadings of items were not included (Steenkamp and Baumgartner, 1998). An overview of descriptive statistics, Cronbach’s alpha, and bivariate correlations of the three dimensions is reported in Table 6.

**TABLE 5:** Pattern matrix, exploratory factor analysis, principal axis, oblimin rotation, 15 items.

**Item label**	**Factor loading^a^**			
	**Destination-oriented**	**Socio-cultural**	**Travel arrangement**	**Communality**
O1	−0.023	0.293	**0.322**	0.248
O2	−**0.604**	−0.153	0.062	0.358
O3	0.028	**0.681**	−0.016	0.368
O4	0.198	**0.491**	−0.041	0.264
O5	0.027	0.061	**0.490**	0.141
O6	−**0.550**	−0.062	−0.026	0.308
O7	−0.154	0.218	**0.248**	0.191
O8	−**0.707**	0.002	−0.032	0.407
O9	−0.152	**0.510**	0.076	0.233
O10	−**0.600**	0.122	−0.084	0.320
O11	−**0.768**	−0.073	0.076	0.457
O12	0.050	−0.065	**0.456**	0.126
O14	0.058	**0.579**	0.027	0.317
O15^b^	0.313	−0.084	**0.351**	0.239
O16	0.027	**0.727**	−0.050	0.408
Eigenvalue	2.430	2.140	1.027	

*KMO > 0.7. Barlett’s test of sphericity was significant at *p* < 0.001. Rotation converged in six iterations. ^*a*^Boldface indicates factor where item is assigned. ^*b*^Item that was reverse coded for the analyses.*

**TABLE 6:** Descriptive statistics, Cronbach’s alpha, and bivariate correlations.

	**Items**	***M***	***SD***	**α**	**1.**	**2.**	**3.**
1. Destination-oriented	O2, O6, O8, O10, O11	3.26	1.12	0.77	–		
2. Socio-cultural	O3, O4, O9, O14, O16	4.60	1.04	0.73	−0.09	–	
3. Travel arrangement	O1, O5, O7, O12, O15	3.88	0.94	0.50	0.08	0.27	–

*Destination-oriented: high mean indicates preference for familiarity in choosing destinations. Socio-cultural: high mean indicates interest for contact with local culture and people. Travel arrangement: high mean indicates preference for avoiding institutionalized arrangements. Correlations are significant at *p* < 0.001.*

### Cluster Analyses

Distinct clusters were uncovered using a multistep cluster analysis with individual scores on the three dimensions as inputs. We used a simplified version of the procedure recommended by Dolnicar and Leisch (2003). The respondents were randomly split into an analysis sample and a validation sample. We used the non-hierarchical K-means method on the analysis sample to identify an initial optimal solution that was next used as cluster seeds for a restricted analysis in the validation sample. The constrained solution was then compared to an unconstrained solution in the same sample; the fit was evaluated with the validity coefficient Kappa. This procedure was repeated for solutions with two to six clusters. Kappa values for the two-, three-, four-, five-, and six-cluster solutions were 0.57, 0.81, 0.74, 0.99, and 0.62 respectively. The high value for the five-cluster solution suggested that these clusters were homogenous and distinct from each other. A discriminant analysis with the five clusters as the dependent variable and dimensions as the independent variables correctly classified 98% of the cases. Thus, individual preferences on the three dimensions yield a high degree of convergence and cohesion along the five preference clusters (see Table 7).

**TABLE 7:** Mean differences in travel preferences by cluster.

	**Preference segment (cluster)**					**ANOVA**		
	**1.**	**2.**	**3.**	**4.**	**5.**	***M***	***F***	***p***
**Travel preferences**								
Destination-oriented	4.57	4.19	2.39	2.67	2.56	3.26	860.01	<0.001
Socio-cultural	5.37	4.02	5.32	5.36	3.48	4.60	707.72	<0.001
Travel arrangement	4.74	3.65	4.66	3.07	3.40	3.88	417.82	<0.001
Number of cases per configuration	277	568	459	312	402			
	Individual mass tourist^a^	Organized mass tourist^a^	Drifter^a^	Explorer^a^	Lone explorer^b^			

*All mean values in clusters differ significantly from the overall average. The overall ANOVA was performed with configurations as independent variable, testing the univariate hypothesis that the means of destination-oriented dimension, socio-cultural dimension, and travel arrangement dimension are equal across preference segments. ^*a*^Suggested correspondence to Cohen (1972). ^*b*^Suggested addition to Cohen (1972).*

Table 7 also reveals that the five clusters differ significantly along each dimension. Cluster 1 is high on the destination-oriented, the socio-cultural, and the travel arrangement dimension, corresponding to the “individual mass tourist”. Cluster 2 is high on the destination-oriented dimension, and low on the socio-cultural and travel arrangement dimensions, which corresponds to the preferences of the “organized mass tourist”. Cluster 3 is low on the destination-oriented dimension but high on the socio-cultural and travel arrangement dimensions, which is similar to the “drifter”. Cluster 4 is similar to the aforementioned in the sense that it is low on the travel-oriented dimension, which makes it correspond to the “explorer”. Cluster 5 scores fairly low on all three dimensions; this type appears to be similar to the previous, albeit with no strong preferences for social contact with locals and cultural immersion. We suggest labeling this additional group as the “lone explorer”, noting that the viability and structure of the group still has to be validated. Each one of the five emerging preference clusters reflect shared perceptions of 14, 28, 23, 16, and 20 percent of the respondents respectively; and even though some clusters are slightly larger than the “organized mass tourist” configuration, each one reflects the shared perceptions of a significant number of tourist.

To test whether the clusters revealed in the analysis can be associated with individual factors, we ran a cross-table with gender as dependent variable and the five clusters as independent. There were no significant gender differences χ^2^(4) = 2.12, *p* = 0.71. We also checked for age differences between the clusters. An analysis of variance suggested that the identified clusters were not closely related to age, *F*(4, 1954) = 9.28, *p* < 0.001, except for the “organized mass tourist” segment being slightly older (*M* = 39.01) and the “explorer” segment slightly younger (*M* = 33.55) than the total average (*M* = 37.92). Table 8 reports the results on whether the continent participants originated from related to the clusters. Though the number of respondents was quite low in some continents, and that not all differences were significant, the table indicates that “explorer” (Cluster 4) and “lone explorer” (Cluster 5) originate in somewhat different proportions from the continents.

**TABLE 8:** Cluster members by continent.

**Continent**	***n***	**Preference segment (cluster)**					
		**1.**	**2.**	**3.**	**4.**	**5.**	
Europe	1326	13.3%	27.5%	23.8%	12.7%	22.8%	100.0%
North America	258	14.0%	29.8%	17.8%	27.9%	10.5%	100.0%
South America	41	12.2%	14.6%	24.4%	39.0%	9.8%	100.0%
Oceania	101	7.9%	27.7%	23.8%	12.9%	27.7%	100.0%
Asia	142	21.1%	28.9%	26.8%	15.5%	7.7%	100.0%
Africa	12	8.3%	33.3%	8.3%	33.3%	16.7%	100.0%

*The overall chi square test indicated significance differences between the preference segments, *χ*^2^(20) = 99.53, *p* < 0.001. Continent subsamples were computed based on information about nationality that was provided by the participants.*

### Further Cluster Validations

If we assume that the clusters based on travel preferences are meaningful, they could relate to valuations of a destination before the trip as well as to perceptions of a destination during the visit. To simplify further analyses, we performed an exploratory factor analysis (principal axis analysis with oblique rotation) on items measuring the initial buying valuations. Item TA1 (affordable price) did not load on any factors, which is why it was decided to remove TA1 from the further analyses. Item TA4 (opportunity to go fishing) formed a single factor, and since this could be a context-specific valuation, we decided to also exclude this item. The remaining items formed three substantial factors that could be labeled “sustainable” (i.e., TA11, TA12, TA13), “nature” (i.e., TA2, TA3, TA6), and “active” (i.e., TA5, TA7, TA8, TA9, TA10). For each factor, average scores were computed with unit weights and no cross loadings; the same factor structure was thereafter used to compute three corresponding average scores for items measuring destination perceptions.

Relationships between the clusters and destination valuations were evaluated with an ANOVA with the valuation of elements of the buying process as dependent variables and the five clusters as the independent variable. The expectation is that preference clusters, because they have different preferences, will value these elements quite differently. A similar set of analysis was run with destination perceptions as dependant variables and the five clusters as the independent variable.

Table 9 shows that although not all differences are significant, destination valuations and destination perceptions tend to vary between preference clusters; however, these differences are comparatively small considering the available response scale options. The same table reports results on associations between the preference clusters and revisit intentions, showing that some clusters differed in their intention to repeat the trip, as well as in their intention to visit the city or the country again.

**TABLE 9:** Mean differences in destination valuations, destination perceptions, and revisit intentions by cluster.

	**Preference segment (cluster)**					**ANOVA results**		
	**1.**	**2.**	**3.**	**4.**	**5.**	***M***	***F***	***p***
**Destination valuations**								
Sustainable	**4.88**	**4.01**	4.27	**4.62**	**3.68**	4.23	35.35	<0.001
Nature	5.73	**5.40**	**5.85**	**5.89**	**5.56**	5.66	15.26	<0.001
Active	**4.71**	**4.01**	4.14	**4.52**	**3.63**	4.14	56.95	<0.001
**Destination perceptions**								
Sustainable	**5.26**	**4.65**	4.86	**5.08**	**4.49**	4.81	19.48	<0.001
Nature	5.90	**5.71**	**6.11**	**6.19**	**5.89**	5.94	12.89	<0.001
Active	5.06	**4.74**	**4.80**	**5.10**	**4.50**	4.81	14.58	<0.001
**Revisit intentions**								
Repeat the trip	**4.09**	3.34	3.61	3.35	**3.14**	3.46	6.14	<0.001
Visit city again	**4.34**	**3.42**	**3.91**	3.60	**3.18**	3.64	10.86	<0.001
Visit country again	**5.00**	**4.33**	**4.84**	4.56	**4.31**	4.57	5.22	<0.001

*Mean values in bold differ significantly from the overall average. Analyses involving revisit intentions included only part of the sample (see section “Materials and Methods”). The overall ANOVA was performed with configurations as independent variable, testing the univariate hypothesis that the means of destination valuations, destination perceptions, and revisit intentions are equal across preference segments.*

## Discussion

The analyzed data failed to invariantly replicate the factor scores from earlier applications of the revised ITR scale (Jiang et al., 2000), which is in line with other studies (Gnoth and Zins, 2010). These small irregularities may signal that the factor structure of the scale is not yet fully developed, or they could be due to contextual or sample-specific processes in the current data. Because the aim was to further opportunities for comparative analyses, and since the analyzed data only weakly suggested a four-factor solution, we chose to stay with a three-factor structure (see Table 5).

Our analyses indicated that based on the three dimensions tapped into by the revised ITR scale, individuals can be meaningfully grouped into clusters that mimic the four types by Cohen (1972). A closer look at the results from the cluster analyses suggested to furthermore distinguishing the “explorer” type into two sub-forms. Both prefer to visit new places, yet one group prefers cultural immersion and to meet and blend with local people, whereas the other group puts much less emphasis on cultural immersion and social contact. This supports previous literature suggesting that dividing the proposed four types further into sub-forms could prove useful to elicit information about more homogenous groups (Uriely, 2009). A next step could be to test if the same clustering pattern emerges among individuals who visit destinations other than currently investigated. Further applications of the revised framework will open for much needed comparable studies, which have the potential to benefit the communication between cooperating stakeholders.

The clustering had a high discriminant validity in that most of the participants clearly belonged to one, and only one of the preference clusters. Nonetheless, these different clusters did not report very different valuations of destination aspects when they initially bought the trip, and only moderate differences in their intentions to return. This seems to suggest that although the tested framework has been proliferating in research, clusters derived from individual preferences for novelty and familiarity are not complete for explaining individual destination choices. An interpretation of these pattern results is that in spite of its intuitive appeal, the present study only establishes weak evidence that the suggested taxonomy has nomological validity at the individual level (Churchill, 1979).

Results from the present study suggested preferential differences between tourists from different continents, in particular between the two segments in our suggested expansion of Cohen’s explorer segment. For example, the proportion of those classified as a “lone explorer” was greater for individuals from the Oceania region than for any other subsample. If it should be the case that the identified preference clusters reflect stable patterns, these might as well relate to many aspects of the tourist experience, of which we have explored only a small fraction. Research in this vein may consider including measures of cultural values to increase the robustness of its findings. There is some indication in the literature that individual scores on dimensions included in the ITR scale can be explained partly through values that are shared by individuals visiting a given destination (Gnoth and Zins, 2010).

Regarding the extent to which broader travel preferences are attached to what aspects tourists emphasize in their vacation, as can be indicated through valuations and perceptions, there was only low predictive validity. This fits to an earlier investigation by Larsen et al. (2011) who found that budget travelers were more likely to agree with statements that ascribe themselves to the “drifter” than mainstream tourists. Although the former were on average more likely to see themselves as more individualistic compared with their mainstream counterpart, both groups were quite similar when it comes to other psychological characteristics. Larsen and colleagues speculated that this might be rooted in the self-perception of backpackers and their socially constructed views of themselves as a group with distinguishing characteristics. Our current findings support this interpretation in the sense that revisit intentions were only to a limited extent (if at all) associated with broader travel preferences, at least when considering the three dimensions included in the revised ITR scale. This ties in well with literature suggesting that having a sole focus on the extent to which individuals seek novelty versus familiarity provides an incomplete picture with regard to understanding the complexity of tourist behavior (Chen et al., 2011).

The revised ITR scale has a potential to reproduce stable clustering for practitioners across time, travelers and contexts, and theoretically meaningful and comparable clusters, but much research remains before unequivocal management recommendations can be made.^3^ In our analyses of the preference-data, we applied the simple K-means clustering technique that is sensitive to sample specific variance. By our rigorous use of estimation samples and validation samples for establishing the number of clusters, as well as the cluster structure, we lowered the risk of sample specific findings. An important fact is that the sample was rather heterogeneous despite that all parts of the data collection took place at the same destination. It contains individuals with different socio-demographic characteristics, that varied in the duration of their traveling, and that were approached at different points during their trip. All this together lowers the probability of sample specific findings, but still, the study will have to be replicated with samples from other destinations to support our present findings.

Forthcoming studies that follow up on our suggestion to replicate the current findings in other destinations could address some limitations of the present investigation. First, given that all constructs were assessed in the same questionnaire, the predictive ability of the clusters could have been inflated by common method variance. We tried to design the questionnaire to limit common method variance by keeping measures of the different constructs well separated in the questionnaire, by wording them differently, by applying different response scales, and to lower method-dependent social desirability issues by letting the participants complete the questionnaire by themselves. Still, these remedies cannot exclude the presence of common method variance in the data (Podsakoff et al., 2012). Future studies are well advised to use different methods for assessing behavior associated with each cluster to get reliable and valid estimates of the associations (Podsakoff et al., 2003). Second, it is because of the cross-sectional design of the current study that we cannot make any conclusion about whether the clustering based on individual scores of the revised ITR scale remains stable across time. If such stability can be demonstrated for at least certain time periods, an interesting future development would be to predict future buying behavior within these periods. Third, forthcoming studies would benefit from broadening the scope toward measures of actual buying behavior since intentions to return are an imperfect predictor for future destination choices (McKercher and Tse, 2012). Fourth, Cronbach’s alpha was acceptable for the destination-oriented (α = 0.77) and socio-cultural dimension (α = 0.73) but poor for the travel arrangement dimension (α = 0.50), based on conventional guidelines (see Streiner, 2003). This represents a noteworthy limitation to the present investigation as clustering that relies upon aggregated measures on each of the three dimensions may lead to biased and less precise clusters, which in succession, may pose a threat to the validity of the reported findings.

## Conclusion

This paper shows that findings from earlier applications of the revised ITR scale replicate to a certain extent in the current sample; the three main dimensions of the scale are with a few modifications reproduced. Further analyses show that the tourists can be grouped (clustered) into preference segments based on the main dimensions included in the scale, which in turn appear to be quite similar to the suggested tourist types of Cohen (1972). When comparing data on the importance assigned to specific aspects when buying the trip, or on the extent to which these aspects were offered at the destination, the various preference segments were dissimilar. Since the magnitude of these differences was rather small, preference-based clusters of tourists have nonetheless only to a limited extent different valuations and perceptions; the same seems to be the case when comparing different groups on their revisit intentions.

It is important to note that belonging to one particular preference cluster may not be unequivocally stable, just as tourist roles might develop and change over time (Gibson and Yiannakis, 2002). To get a deeper understanding of the psychological processes that take part in shaping tourist experiences, future research could extend the scope beyond theoretically developed taxonomies as employed in the present case. It seems plausible that the extent by which initial valuations correspond with perceptions during the trip constitutes another aspect that feeds into how a destination is experienced, and related decisions, potentially more so than whether individuals report to prefer more (or less) novelty (or familiarity) if traveling away from home. A more detailed discussion on how a psychological approach can be a useful starting point for social scientific enquiries on tourist experiences is provided by Larsen (2007) as well as Larsen et al. (2017).

## Data Availability Statement

The datasets generated for this study are available on request to the corresponding author.

## Ethics Statement

This work complied with the general guidelines for research ethics by the Norwegian National Committees for Research Ethics in the Social Sciences and the Humanities (NESH). Formal approval by an ethics committee was not required as per applicable institutional guidelines and regulations.

## Author Contributions

RD, SL, and KW contributed to the conception, design, and data collection of the study. TØ performed the statistical analysis. TØ and RD wrote the first draft of the manuscript. All authors contributed to the manuscript revision, read, and approved the submitted version.

## Conflict of Interest

The authors declare that the research was conducted in the absence of any commercial or financial relationships that could be construed as a potential conflict of interest.

## Footnotes

1 The assumption that novelty seeking is an important factor underlying individual travel decisions can also be found in the social psychological model of tourism motivation (Iso-Ahola, 1982), the psychographic approach (Plog, 2001), and the travel career pattern approach (Pearce and Lee, 2005) amongst others.

2 The reported number only refers to respondents who were 18 years and over at the time of the data collection. Total percentages are not 100 for each of the listed categories due to some missing values.

3 Note that our empirical development of the preference clusters was based on the revised ITR scale, which is but one of a number of theoretically founded alternatives (e.g., Yiannakis and Gibson, 1992; Keng and Cheng, 1999).
